# Diagnostic value of somatosensory evoked potentials for paroxysmal sympathetic hyperactivity: a retrospective cohort study

**DOI:** 10.3389/fneur.2026.1699872

**Published:** 2026-01-28

**Authors:** Lizhi Liu, Yuqing Han, Hui Feng, Huiyue Feng, Fangyu Chen, Juanjuan Fu

**Affiliations:** Department of Rehabilitation Medicine, The Affiliated Jiangning Hospital of Nanjing Medical University, Nanjing, China

**Keywords:** Coma Recovery Scale-Revised score, diagnosis, disorders of consciousness, paroxysmal sympathetic hyperactivity, somatosensory evoked potentials

## Abstract

**Purpose:**

This study aimed to investigate the diagnostic value of somatosensory evoked potentials (SEPs) in patients with paroxysmal sympathetic hyperactivity (PSH) and to identify independent predictors of the condition.

**Methods:**

A retrospective cohort study was conducted on 123 patients with prolonged disorders of consciousness (PDOC) admitted to the Critical Care Rehabilitation Department of Nanjing Jiangning Hospital between August 2022 and August 2024. Patients were classified into PSH-positive (PSH+) and PSH-negative (PSH−) groups according to the Paroxysmal Sympathetic Hyperactivity Assessment Measure (PSH-AM). Demographic, clinical, and SEPs parameters were collected. Univariate and multivariate logistic regression analyses were employed to examine the association between these variables and PSH. The predictive performance was evaluated using receiver operating characteristic (ROC) curve analysis.

**Results:**

A total of 123 patients with prolonged disorders of consciousness were enrolled in the study. Among these, 34 patients (27.64%) were classified into the PSH + group and 89 patients (72.36%) into the PSH − group. Multivariate logistic regression analysis identified younger age (OR = 0.96, 95% CI: 0.92–0.99, *p* = 0.02), male patient (OR = 0.28, 95% CI: 0.09–0.75, *p* = 0.02), and reduced N20-P25 amplitude (OR = 0.34, 95% CI: 0.13–0.70, *p* = 0.01) as independent predictors of PSH. Using a cut-off value of 1.19 μV for the N20-P25 amplitude, the area under the curve (AUC) for discriminating PSH was 0.811 (95% CI: 0.71–0.912), yielding a sensitivity of 79.7% and a specificity of 75%. The combination of these three predictors improved the AUC to 0.846 (95% CI: 0.75–0.942). After adjusting for potential confounders, partial correlation analysis demonstrated a significant negative correlation between N20-P25 amplitude and PSH-AM score (adj. *r* = −0.30, *p* = 0.003).

**Conclusion:**

A reduced N20-P25 amplitude may serve as an independent and objective electrophysiological biomarker for the early prediction of PSH. In combination with younger age and male patient, it contributes to the identification of high-risk populations and offers valuable guidance for clinical management.

## Introduction

Paroxysmal sympathetic hyperactivity (PSH) is a clinical syndrome that typically occurs following acute severe brain injury and is characterized by episodic adrenergic crises. Its manifestations include fever, tachycardia, hypertension, tachypnoea, profuse sweating, and dystonia (1, 2). This condition has previously been described under various terminologies, such as sympathetic storm, diencephalic epilepsy, midbrain dysregulation syndrome, and paroxysmal autonomic instability with dystonia (3). The reported incidence of PSH varies across studies, ranging from 7.7 to 33% following traumatic brain injury (TBI) (4–7). TBI represents the most common etiology of PSH, followed by hypoxic brain injury, with stroke being the third most frequent cause (4). Beyond representing a pathophysiological phenomenon, PSH is consistently associated with a range of adverse clinical outcomes. Studies have demonstrated that patients with PSH exhibit poorer Glasgow Outcome Scale scores and lower Functional Independence Measure results (7–10). Additionally, these patients often require prolonged mechanical ventilation, extended stays in intensive care unit and overall hospitalization, and are subject to higher rates of infection, increased tracheostomy requirements, and significantly elevated healthcare costs (6, 10–12). The mechanisms underlying its poor prognosis are multifactorial: recurrent symptomatic episodes and complex treatment needs contribute to extended hospital stays; PSH-related fever may exacerbate secondary brain injury (13); and autonomic dysfunction may also impair neurorecovery by modulating inflammatory responses (14, 15).

Currently, the diagnosis of PSH relies primarily on clinical assessment tools such as the PSH Assessment Measure (PSH-AM) (16–19). The absence of objective biomarkers can lead to delayed diagnosis, under-recognition, or misdiagnosis as other conditions, including sepsis, epilepsy, or withdrawal syndromes (2, 20–22), potentially resulting in treatment delays. This underscores the urgent need to develop objective evaluation tools. The pathophysiology of PSH remains incompletely understood. The predominant model remains the “disconnection theory” (23), which posits that brainstem excitatory centres are released from higher inhibitory control, leading to a hypersympathetic state. Recent neuroimaging studies suggest that PSH is closely associated with injuries to deep brain structures, such as the diencephalon, dorsolateral midbrain, and upper pons, as well as to periventricular white matter and the corpus callosum, particularly in patients with diffuse axonal injury or bilateral diencephalic lesions (24–28). These regions are critical for sensory transmission and autonomic regulation; their damage may lead to aberrant amplification of non-noxious stimuli, triggering excessive sympathetic responses. However, conventional CT or MRI often lacks the sensitivity required to fully assess such microstructural and functional connectivity impairments, especially during the acute phase. Hence, there is a pressing clinical need for electrophysiological techniques capable of providing an objective and quantitative evaluation of the functional integrity of relevant neural pathways.

Somatosensory evoked potentials (SEPs) quantitatively assess the functional integrity of the sensory pathway by recording electrophysiological responses within the central nervous system following peripheral nerve stimulation. This pathway extends from peripheral nerves through the spinal cord, brainstem, thalamus, and up to the cerebral cortex (29). SEPs serve as a vital tool in neurocritical care for prognostic evaluation (30–32). The bilateral absence of the cortical N20 potential is widely recognized as a reliable predictor of poor neurological outcome after severe brain injury (33–35). Notably, the thalamocortical radiation and cortical sensory areas involved in SEP generation (29) exhibit anatomical and functional convergence with diencephalic brainstem autonomic pathways implicated in PSH. We therefore hypothesize that SEP abnormalities may reflect the extent of damage to neural structures relevant to the development of PSH, thereby offering an objective basis for early identification.

To date, the potential utility of SEPs in the management of PSH remains largely unexplored. To address this gap, we conducted a retrospective clinical study. This study aimed to elucidate the relationship between early SEP parameters and the occurrence of PSH, with the goal of providing electrophysiological evidence to support early objective diagnosis and intervention for this condition.

## Methods

### Patients and study design

This retrospective observational study enrolled 123 patients with prolonged disorders of consciousness (PDOC) who were admitted to the Critical Care Rehabilitation Department of Nanjing Jiangning Hospital between August 2022 and August 2024. The cohort comprised 79 male and 44 female patients. Inclusion criteria were as follows: (1) age between 18 and 75 years; (2) disease duration of 28 to 90 days; (3) diagnosis of hypoxic-ischaemic encephalopathy, cerebral haemorrhage, or severe traumatic brain injury; (4) diagnosis of a vegetative state/unresponsive wakefulness syndrome (VS/UWS) or minimally conscious state (MCS), established through five consecutive weekly Coma Recovery Scale-Revised (CRS-R) assessments (36, 37); and (5) absence of effects from sedatives, anaesthetics, or muscle relaxants. Exclusion criteria included: (1) Pre-existing structural brain injury (e.g., prior stroke, traumatic brain injury, or neurosurgical intervention) before the current acute brain injury event, or a history of neurodegenerative conditions; (2) Concurrent diseases known to affect autonomic nervous system function, including but not limited to: thyroid dysfunction, diabetes mellitus with peripheral neuropathy, autoimmune dysautonomia (e.g., pure autonomic failure, multiple system atrophy), spinal cord injury above the thoracic level, significant cardiac arrhythmia, renal failure, hepatic encephalopathy, and primary neurodegenerative disorders (e.g., Parkinson’s disease, multiple sclerosis). All patients were screened through detailed medical history, clinical examination, and indicated laboratory or neurophysiological tests to exclude these conditions; (3) clinically unstable conditions, including hemodynamic instability, severe respiratory failure, or acute hydrocephalus; (4) excessive artifacts in SEP recordings; or (5) abnormal peripheral (N9) or cervical (N13) SEP components.

Informed written consent was obtained from the legal surrogates of all participants prior to enrolment. The study was approved by the Ethics Committee of Jiangning Hospital Affiliated to Nanjing Medical University (Approval No: 2022-03-047-k01).

### Sample size calculation

Based on prior studies reporting a PSH incidence of approximately 25–35% in patients with prolonged disorders of consciousness (8, 19), we estimated that a sample size of at least 120 patients would be required to detect a significant difference in SEP parameters between groups, with a two-sided *α* of 0.05 and a power (1–*β*) of 80%. The final sample of 123 patients met this target.

### Data collection

Baseline information was retrospectively collected for all patients with PDOC. Demographic and baseline clinical characteristics: Age, sex, time since brain injury, duration of intensive care unit (ICU) stay, history of decompressive craniectomy, smoking history, alcohol consumption, and history of hypertension, diabetes, and coronary heart disease.

### Etiology and neuroimaging findings

The primary etiology (hypoxic-ischaemic encephalopathy, cerebral haemorrhage, or severe traumatic brain injury) was determined based on admission neuroimaging. Hydrocephalus was assessed using the Evans index (EI), an objective imaging indicator (38). The EI was calculated as the ratio of the maximum width between the anterior horns of the bilateral lateral ventricles to the maximum internal transverse diameter of the skull at the same level, based on initial cranial computed tomography axial images obtained after admission. An EI greater than 0.31 was used as the diagnostic criterion for hydrocephalus, while an EI ≤ 0.31 indicated absence of hydrocephalus (38, 39). All imaging measurements were performed independently by two physicians who were blinded to patient group assignments. Any discrepancies were resolved through discussion to ensure objectivity.

### Laboratory indicators

Fasting venous blood samples were collected from patients between 06:00 and 08:00 within 48 h of admission to measure plasma cortisol (COR) and adrenocorticotropic hormone (ACTH) concentrations.

### Clinical scale scores

The diagnosis of PSH was based on the PSH-AM scale (16). This instrument consists of two components: the Clinical Feature Scale (CFS) and the Diagnosis Likelihood Tool (DLT). The CFS assesses the severity of six clinical features of PSH: heart rate, respiratory rate, systolic blood pressure, temperature, sweating, and posturing. Each feature is rated on a scale from 0 to 3. The DLT addresses diagnostic specificity through 11 binary features, each scored 1 point if present. The total PSH-AM score was calculated as the sum of the CFS and DLT scores. Patients were classified according to the following criteria: a score below 8 was considered unlikely to indicate PSH; a score between 8 and 16 indicated possible PSH; and a score of 17 or higher indicated probable PSH (16). For the purposes of this study, patients with a total score of 17 or higher were classified as PSH+, while those scoring below this threshold were classified as PSH−. Two neurologists, blinded to patient identifiers and all other clinical information, independently reviewed medical records to assign PSH-AM scores. Any discrepancies between their assessments were resolved through discussion until consensus was reached.

Neurologists experienced in the evaluation of patients with PDOC performed the CRS-R assessments. For each patient, the CRS-R was administered daily over five consecutive days during the first week after admission. The CRS-R consists of 23 items grouped into six subscales: auditory, visual, motor, oromotor/verbal, communication, and arousal functions. It is regarded as the most reliable diagnostic tool for distinguishing between VS/UW and MCS (36, 40). Total scores on the CRS-R range from 0 to 23.

### Neuroelectrophysiological data

Somatosensory Evoked Potentials (SEPs) were recorded within 1 week of admission to the Critical Care Rehabilitation Department, in accordance with standard procedures (29), using a Neuron-Spectrum-4 electromyography and evoked potential instrument (Neurosoft, Russia). Electroencephalography electrodes were placed according to the international 10–20 system. Silver chloride (AgCl) electrodes were used to record SEPs at Erb’s point (N9), the seventh cervical spinous process (N13), and the scalp (CP3/CP4), with a reference electrode positioned at the midfrontal forehead (Fz). Skin-electrode impedance was maintained below 5 kΩ. The median nerve was stimulated using a bipolar transdermal electrical stimulator placed at the wrist. Stimuli consisted of 0.1 ms monophasic square waves, with intensity adjusted to elicit consistent muscle twitching in the thenar region. Five hundred trials were performed at a rate of 2 Hz, using a bandpass filter of 2–2000 Hz. Two independent clinical neurophysiologists, who were blinded to patient conditions and PSH-AM scores, performed all SEP recordings. Responses were excluded if they lacked bilateral reproducibility or if noise interference compromised the interpretation of cortical SEPs. Bilateral absence was classified only when all cortical recordings demonstrated noise levels below 0.25 μV and non-reproducible cortical potentials. For reproducible cortical potentials (occurring at least 4.5 ms after N13), triplicate measurements of N20–P25 amplitudes were conducted. Amplitudes were rounded to two decimal places, and the highest amplitude from three consecutive cortical recordings was selected for each side.

### Statistical analysis

Data were analysed using IBM SPSS Statistics, Version 22.0 (IBM Corp., Armonk, NY, USA). Descriptive statistics were used to characterize the sample in terms of demographic and clinical variables. The Shapiro–Wilk test was employed to assess data normality. Continuous variables with a normal distribution were reported as means ± standard deviations (SD), while skewed variables were summarized as medians with interquartile ranges (25th–75th percentiles). Categorical variables were expressed as frequencies and percentages. The Mann–Whitney U test and independent samples t-tests were used, as appropriate, to compare demographic, clinical, and SEP parameters between groups. Pearson’s chi-square test was used to compare categorical SEP variables between the PSH + and PSH − groups. A multivariate logistic regression model was constructed, incorporating SEPs parameters along with clinical predictors including age, disease duration, intensive care unit stay duration, lesion location, craniectomy status, and Coma Recovery Scale-Revised score. Receiver operating characteristic (ROC) curve analyses were conducted to establish outcome thresholds for SEP amplitudes. Optimal amplitude thresholds were determined by maximizing the Youden index (*J* = sensitivity + specificity−1). Partial correlation analysis was used to assess the relationships between PSH-AM scores and SEP parameters, with adjustment for baseline confounding factors. Statistical significance was defined as a two-sided *p* value < 0.05 for all analyses.

## Results

**Figure 1:**
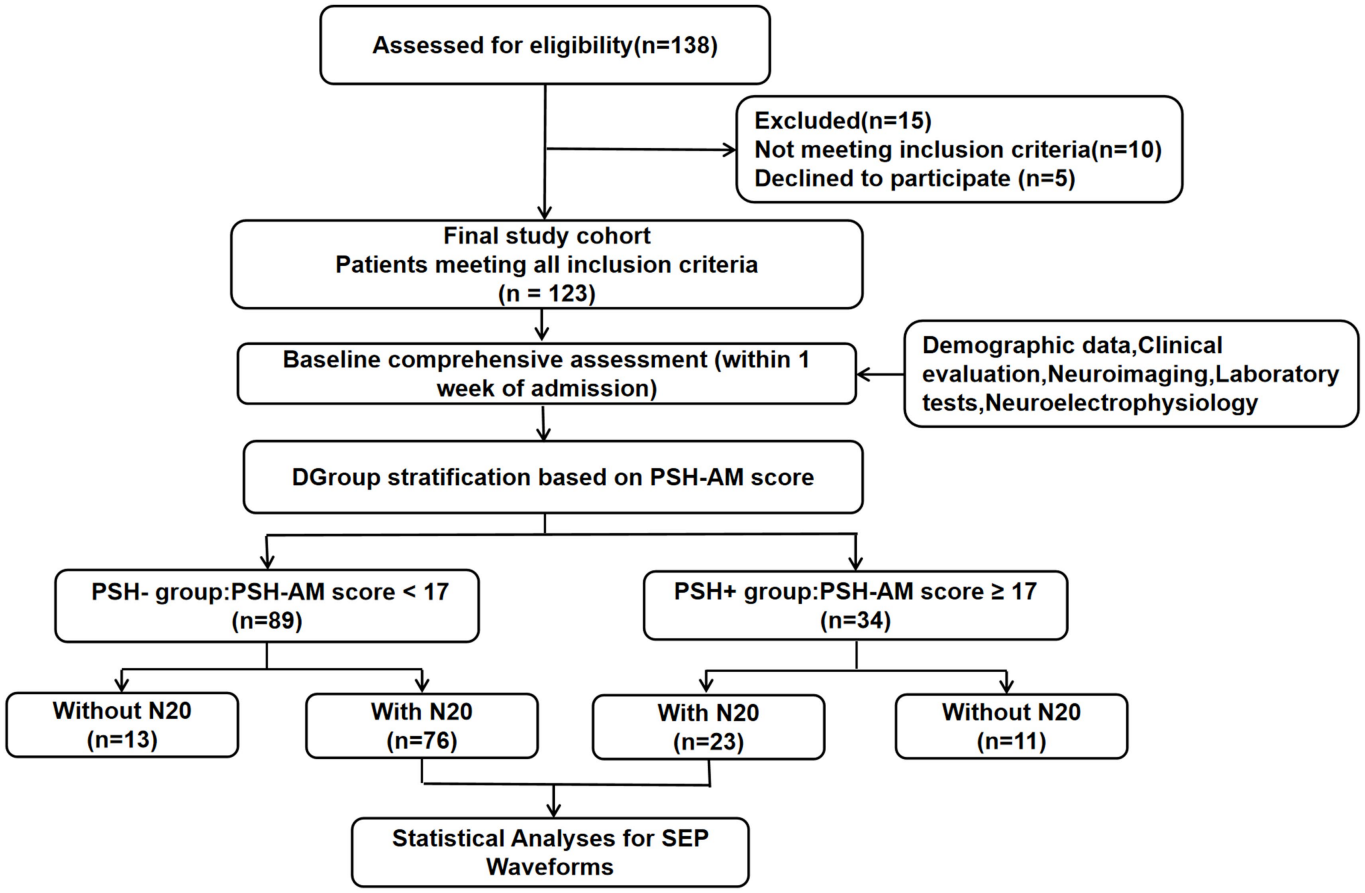
Flow diagram illustrating the patient screening and exclusion criteria. PSH, paroxysmal sympathetic hyperactivity; CRS-R, Coma Recovery Scale–Revised.

The baseline assessment in this study was conducted at an average of 43.60 days following brain injury. A total of 123 patients with prolonged disorders of consciousness were enrolled, comprising 34 patients (27.64%) in the PSH + group and 89 patients (72.36%) in the PSH – group (Figure 1). Compared with the PSH − group, patients in the PSH + group were significantly younger (53.15 ± 14.84 years vs. 60.38 ± 11.68 years; *p* = 0.01) and had a higher proportion of male patient (*p* = 0.03). No significant differences were observed between the two groups in terms of disease duration, length of intensive care unit stay, etiology, smoking history, alcohol history, history of hypertension, diabetes, coronary heart disease, decompressive craniectomy, Evans index, hydrocephalus, cortisol levels, or adrenocorticotropic hormone levels (all *p* > 0.05). Baseline assessment indicated that the proportion of patients in a VS/UWS was significantly higher in the PSH + group than in the PSH − group (*p* = 0.02). Additionally, Coma Recovery Scale–Revised (CRS–R) scores were lower in the PSH + group (8.88 ± 4.29) compared to the PSH − group (10.48 ± 3.85; *p* = 0.05) (Table 1).

**Table 1:** Baseline characteristics of patients.

Variable		Total	PSH − group	PSH + group	*p* value
Sex	Male	79 (64.2%)	52 (58.4%)	27 (79.4%)	0.03*
	Female	44 (35.8%)	37 (41.6%)	7 (20.6%)	
Mean age (years)		58.38 ± 12.98	60.38 ± 11.68	53.15 ± 14.84	0.01^*^
Disease duration (days)		43.60 ± 15.51	44.26 ± 16.24	41.88 ± 13.47	0.45
ICU Stay (days)		21.93 ± 9.24	21.83 ± 8.76	22.21 ± 10.54	0.84
Etiology	Stroke	85 (69.1%)	64 (71.9%)	21 (61.8%)	0.27
	Traumatic brain injury	24 (19.5%)	16 (18%)	8 (23.5%)	
	Hypoxic-ischaemic encephalopathy	14 (11.4%)	9 (10.1%)	5 (14.7%)	
Craniectomy	NO	62 (50.4%)	44 (49.4%)	18 (52.9%)	0.73
	YES	61 (49.6%)	45 (50.6%)	16 (47.1%)	
Smoking	NO	59 (48%)	45 (50.6%)	14 (41.2%)	0.35
	YES	64 (52%)	44 (49.6%)	20 (58.8%)	
Alcohol drinking	NO	63 (51.2%)	47 (52.8%)	16 (47.1%)	0.57
	YES	60 (48.8%)	42(47.2%)	18(52.9%)	
Hypertension	NO	29 (23.6%)	20 (22.5%)	9 (26.5%)	0.64
	YES	94 (75.4%)	69 (77.5%)	25 (73.5%)	
Diabetes	NO	102 (82.9%)	75 (84.3%)	27 (79.4%)	0.52
	YES	21 (17.1%)	14 (15.7%)	7 (20.6%)	
Coronary heart disease	NO	107 (87%)	76 (85.4%)	31 (91.2%)	0.39
	YES	16 (13%)	13 (14.6%)	3 (8.8%)	
Hydrocephalus	NO	63 (51.2%)	49 (55.1%)	14 (41.2%)	0.17
	YES	60 (48.8%)	40 (40.9%)	20 (58.8%)	
Cortisol (nmol/L)		472.81 ± 158.79	456.76 ± 152.35	514.32 ± 169.72	0.07
Adrenocorticotropic hormone (pg/mL)		47.42 ± 24.97	46.42 ± 25.87	50.01 ± 22.67	0.09
Ewans Index		0.29 ± 0.06	0.29 ± 0.06	0.31 ± 0.07	0.20
PSH-AM Score		9.96 ± 5.36	7.20 ± 3.28	16.74 ± 2.81	<0.001^***^
CRS-R Score		10.04 ± 4.02	10.48 ± 3.85	8.88 ± 4.29	0.05
Diagnosis	Vegetative state/Unresponsive wakefulness syndrome	45 (36.6%)	27 (30.3%)	18 (52.9%)	0.02^*^
	Minimally conscious state	78 (63.4%)	62 (69.7%)	16 (47.1%)	

Data are presented as mean ± standard deviation or number (%) as appropriate. Hydrocephalus was defined as an Evans Index (EI) > 0.31. PSH, paroxysmal sympathetic hyperactivity; PSH-AM, Paroxysmal Sympathetic Hyperactivity Assessment Measure, Coma Recovery Scale–Revised; ICU, intensive care unit. Statistical significance: *p < 0.05, **p < 0.01, ***p < 0.001.

Compared with patients in the PSH − group, a significantly higher proportion of those in the PSH + group exhibited bilateral absence of the N20 potential (*p* = 0.03). Among the 34 patients in the PSH + group, 11 (32.4%) showed bilateral absence of N20, while 23 (67.6%) exhibited detectable N20 responses. The PSH + group demonstrated significantly prolonged N20 latency compared to the PSH − group (22.08 ± 3.42 ms vs. 20.63 ± 2.91 ms; *p* = 0.04). Additionally, the N20–P25 amplitude was significantly lower in the PSH + group than in the PSH − group (1.11 ± 0.78 μV vs. 2.60 ± 1.86 μV; *p* < 0.001) (Table 2).

**Table 2:** Somatosensory evoked potential in patients between PSH− and PSH + group.

Variable		Total	PSH − group	PSH + group	*p* value
A. N20 response (*n* = 123)					
N20 Response	Bilateral absence	24 (19.5%)	13 (14.6%)	11 (32.4%)	*0.03* ^*^
	Any presence	99 (80.5%)	76 (85.4%)	23 (67.6%)	
B. N20-P25 waveform parameters (*n* = 99)					
N20 latency, ms		20.97 ± 3.08	20.63 ± 2.91	22.08 ± 3.42	0.04^*^
N20-P25 amplitude, μV		2.25 ± 1.78	2.60 ± 1.86	1.11 ± 0.78	<0.001^***^

Data are presented as mean ± standard deviation or number (%). The N20-P25 amplitude and N20 latency were analyzed only for patients with elicitable cortical N20 responses (*n* = 99). PSH, paroxysmal sympathetic hyperactivity. Statistical significance: *p < 0.05, **p < 0.01, ***p < 0.001.

To evaluate the role of SEP parameters in distinguishing PSH, we performed a logistic regression analysis using PSH group assignment (PSH − or PSH+) as the dependent variable. In the univariate logistic regression analysis, we assessed the association between various baseline variables and the risk of PSH. The results indicated that younger age (OR = 0.96, *p* = 0.01) and male patient (OR = 0.35, *p* = 0.03) were significantly associated with an increased risk of PSH. Among the SEP parameters, reduced N20–P25 amplitude (OR = 0.28, *p* < 0.001) and prolonged N20 latency (OR = 1.14, *p* = 0.06) also showed notable associations. In contrast, variables such as etiology, disease duration, length of intensive care unit stay, decompressive craniectomy, and hydrocephalus did not demonstrate statistically significant associations (all *p* > 0.05). Based on these findings, variables with *p* values < 0.1 were included in the subsequent multivariate logistic regression analysis. The multivariate model confirmed that younger age (OR = 0.96, *p* = 0.02), male sex (OR = 0.28, *p* = 0.02), and reduced N20–P25 amplitude (OR = 0.34, *p* = 0.01) were independent predictors of PSH (Table 3).

**Table 3:** Multivariate logistic regression analysis of somatosensory evoked potential for the PSH diagnosis in patients.

Variable	Univariate analysis		Multivariate analysis	
	OR (95% CI)	*p* value	OR (95% CI)	*p* value
Age	0.96 (0.93–0.99)	0.01^*^	0.96 (0.92, 0.99)	0.02^*^
Sex	0.36 (0.13–0.89)	0.03^*^	0.28 (0.09, 0.75)	0.02^*^
Disease duration (days)	0.99 (0.96–1.02)	0.45		
Etiology	1.69 (0.48–5.49)	0.39		
Craniectomy	0.87 (0.39–1.92)	0.73		
Hydrocephalus (yes)	1.75 (0.79–3.96)	0.17		
CRS-R Score	0.90 (0.81–1.00)	0.05	1.03 (0.83, 1.28)	0.82
N20 classification (present)	0.36 (0.14–0.91)	0.03^*^	0.72 (0.03, 27.62)	0.85
N20 latency	1.14 (1.00–1.33)	0.06	0.99 (0.83, 1.18)	0.93
N20-P25 amplitude	0.28 (0.12–0.54)	0.001^**^	0.34 (0.13, 0.70)	0.01^*^
Cortisol	1.00 (1.00–1.01),	0.08	1.00 (1.00, 1.006)	0.14
Adrenocorticotropic hormone	1.01 (0.99–1.02)	0.48		

The model included variables with *p* < 0.1 from univariate analysis. PSH, paroxysmal sympathetic hyperactivity; OR, odd ratio; CI, confidence interval; CRS-R, coma recovery scale-revised; Statistical significance: *p < 0.05, **p < 0.01, ***p < 0.001.

**Figure 2:**
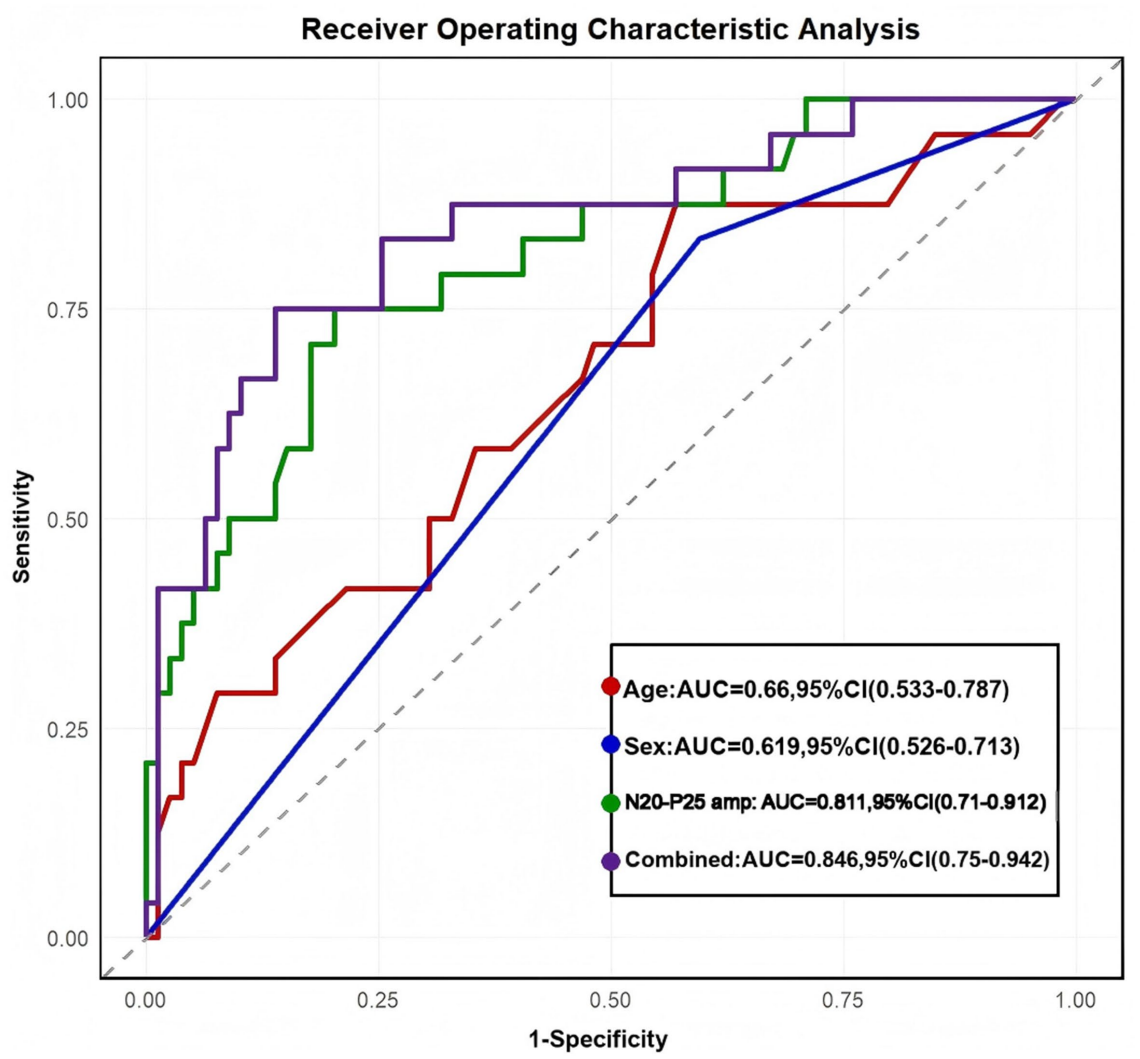
ROC analysis for the PSH diagnosis. The area under the ROC curve (AU-ROC) for age, sex (coded male as 1, vs. female as 0), N20–P25 amplitude, and combination (three predictors) was 0.66 (95% CI: 0.533–0.787), 0.619 (95% CI: 0.526–0.713), 0.811 (95% CI: 0.71–0.912), and 0.846 (95% CI: 0.75–0.942), respectively. PSH, Paroxysmal Sympathetic Hyperactivity.

The optimal diagnostic threshold for age was determined to be 64.5 years, yielding an AUC-ROC of 0.66 (95% CI:0.533–0.787), with a sensitivity of 43% and a specificity of 87.5%. For sex (coded male as 1, vs. female as 0), the model achieved an AUC-ROC of 0.619 (95% CI: 0.526–0.713), with a sensitivity of 41% and a specificity of 83.3%. The N20–P25 amplitude demonstrated stronger predictive performance at a cut-off value of 1.19 μV, yielding an AUC-ROC of 0.811 (95% CI:0.71–0.912), a sensitivity of 79.7%, and a specificity of 75%. Notably, the combination of all three predictors resulted in further improved discrimination, achieving an AUC-ROC of 0.846 (95% CI:0.75–0.942), with a sensitivity of 82.3% and a specificity of 81.2% (Figure 2).

**Figure 3:**
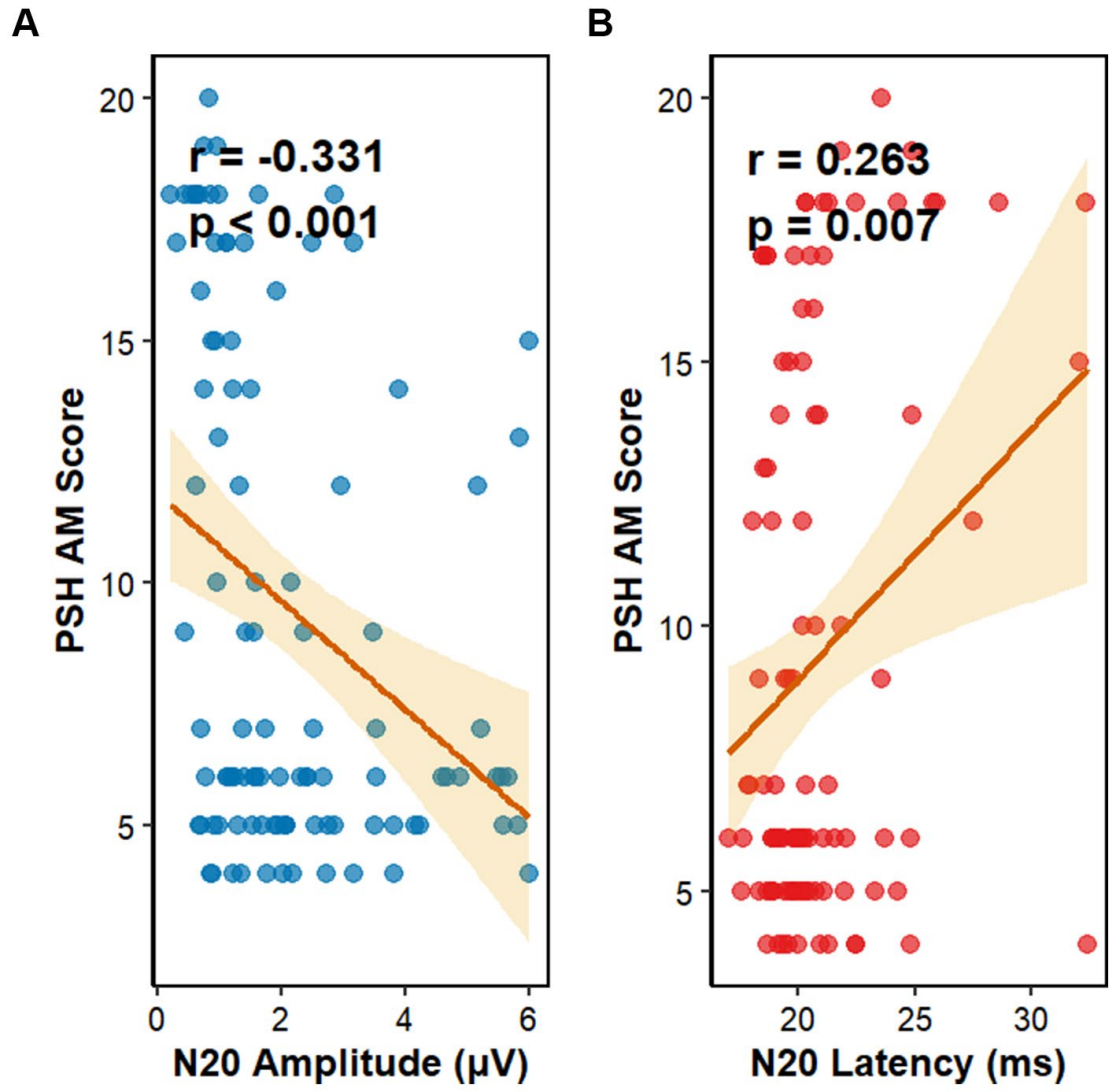
Scatter plots showing unadjusted correlations between Paroxysmal Sympathetic Hyperactivity Assessment Measure (PSH-AM) scores and somatosensory evoked potential parameters. **(A)** Negative correlation between N20–P25 amplitude and PSH-AM score (*r* = −0.33, *p* < 0.001). **(B)** Positive correlation between N20 latency and PSH-AM score (*r* = 0.26, *p* = 0.007). The corresponding partial correlation coefficients, adjusted for age, sex, disease duration, ICU stay, lesion location, and craniectomy status, are reported in the results section (adjusted *r* = 0.21, *p* = 0.04 for latency; adjusted *r* = −0.30, *p* = 0.003 for amplitude).

Initially, Pearson correlation analysis was conducted (Figure 3), which revealed a statistically significant negative correlation between N20–P25 amplitude and PSH-AM scores (*r* = −0.33, *p* < 0.001). In contrast, N20 latency showed a positive correlation with PSH-AM scores (*r* = 0.26, *p* = 0.007). Subsequently, partial correlation analysis was performed after controlling for key clinical confounders, including age, sex, disease duration, length of intensive care unit stay, lesion location, and craniectomy status. The results demonstrated that the N20–P25 amplitude remained significantly negatively correlated with PSH-AM scores (adjusted *r* = −0.30, *p* = 0.003), while N20 latency continued to exhibit a positive association with PSH-AM scores (adjusted *r* = 0.21, *p* = 0.04).

## Discussion

This study is the first to investigate the diagnostic value of somatosensory evoked potentials for paroxysmal sympathetic hyperactivity. The key findings are as follows: First, a reduced N20-P25 amplitude (OR = 0.34) was identified as an independent predictor of PSH, demonstrating good discriminative ability (AUC = 0.811). Second, younger age (OR = 0.96) was confirmed as an independent risk factor. Third, male patient (OR = 0.28) also emerged as an independent predictor. These results collectively provide new electrophysiological evidence for the early and objective identification of PSH.

The reduction in N20-P25 amplitude as a biomarker for PSH has a pathophysiological basis. Neuroimaging studies consistently show that PSH is closely associated with diffuse axonal injury in deep brain structures (e.g., diencephalon, brainstem) and white matter pathways connecting the thalamus, cortex, and limbic system (16, 41, 42). These regions are integral components of the SEP signaling pathway, particularly the thalamocortical radiation (29). At the neural structural level, SEP offers direct insight into the integrity of the somatosensory pathway from peripheral nerve stimulation through spinal cord, brainstem, thalamus, and ultimately to the primary somatosensory cortex. This pathway’s functional convergence with autonomic regulatory centers - specifically within the diencephalon and brainstem regions frequently implicated in PSH - provides a mechanistic link between SEP abnormalities and sympathetic hyperactivity. The quantifiable nature of SEP parameters, particularly N20-P25 amplitude, enables objective assessment of thalamocortical pathway function, which is otherwise difficult to evaluate through conventional imaging in acute settings. Consequently, a decrease in SEP amplitude likely reflects the extent of damage to neural structures implicated in the pathogenesis of PSH, suggesting that PSH is not an isolated dysautonomia but rather a component of widespread neural network disruption following severe brain injury. This interpretation is supported by the results of the partial correlation analysis adjusted for confounders in this study: after controlling for age, sex, and other factors, the N20-P25 amplitude remained significantly negatively correlated with the PSH-AM score (adjusted *r* = −0.30, *p* = 0.003). This indicates that a lower amplitude, reflecting more severe damage to the thalamocortical pathway, is associated with more pronounced clinical manifestations of sympathetic hyperactivity. This aligns with previous research suggesting that quantitative analysis of SEP amplitude provides more nuanced prognostic information compared to a simple dichotomous present/absent assessment (43–45). Compared to behavioral scales such as the Coma Recovery Scale-Revised (CRS-R), SEPs provide this direct, pathway-specific assessment of thalamocortical integrity. In this study, the CRS-R score was not an independent predictor of PSH, suggesting that PSH pathophysiology may be more closely linked to damage in specific neural structures (e.g., thalamocortical and autonomic regulatory pathways) rather than to the overall level of behavioral consciousness. Importantly, this does not diminish the clinical value of CRS-R in the comprehensive management of patients with disorders of consciousness. The CRS-R remains the gold standard for diagnosing and differentiating between vegetative state/unresponsive wakefulness syndrome and minimally conscious state, providing essential guidance for neurorehabilitation planning and prognostic counseling. Thus, in the context of PSH assessment, CRS-R and SEPs serve complementary roles: while the CRS-R characterizes the global behavioral phenotype, SEPs offer an objective, pathway-specific biomarker that may enhance early identification of patients at high risk for developing sympathetic hyperactivity. Regarding clinical feasibility, SEP is advantageous due to its non-invasive nature, bedside applicability, and increasing accessibility with portable devices, making it suitable for implementation in ICU settings. However, its reliability requires controlling for potential confounders, including pharmacological influences, technical variables (e.g., electrode impedance, stimulation parameters), and patient-specific factors. By adhering to standardized protocols, these challenges can be mitigated, positioning SEP as a practical and objective tool for early PSH risk stratification and monitoring in neurocritical care.

This analysis reaffirms that younger age (8, 46) and male patient (8, 47, 48) are independent risk factors for PSH. Younger patients showed a higher risk of PSH (mean age: 53.15 ± 14.84 years vs. 60.38 ± 11.68 years; *p* = 0.01), which may be attributed to greater neural plasticity and more reactive sympathetic responsiveness in younger individuals (48). Following injury, the younger brain may retain more intact lower sympathetic pathways, which could become hyperexcitable after losing inhibitory control from higher centers (5, 49). Male patient was also associated with a significantly increased risk of PSH (79.4% vs. 58.4%; *p* = 0.03), and multivariate analysis confirmed its independent predictive value (OR = 0.28). This finding may be related to sex-based differences in autonomic regulation, influenced by hormonal factors (50). Androgens have been shown to enhance sympathetic tone and reduce parasympathetic activity (51), whereas estrogens are thought to confer neuroprotective and anti-inflammatory effects (52). These mechanisms may explain the lower incidence of PSH among female patients.

It is noteworthy that this study did not find the CRS-R score to be an independent predictor of PSH. This result suggests that the occurrence of PSH may be directly related to the severity of damage to specific anatomical structures (26, 53, 54), rather than being linearly correlated with the macro-level of behavioral consciousness. Severe diffuse axonal injury can lead to both low GCS scores and PSH, although a direct causal relationship between the two may not exist. Therefore, compared to global consciousness scales, electrophysiological measures that directly assess the integrity of relevant neural networks, such as SEPs, may offer greater specificity in predicting PSH.

The findings of this study suggest that the value of SEPs, particularly the N20-P25 amplitude, may extend beyond acute diagnosis. Given that PSH symptoms can persist for months, integrating SEP monitoring into neurorehabilitation protocols could serve as an objective tool for assessing neurological recovery trajectory, predicting functional outcomes, and guiding individualized treatment. During the rehabilitation phase, serial SEP assessments may aid in: (1) Monitoring recovery progress: An increase in amplitude may indicate improved function of the thalamocortical pathway; (2) Predicting rehabilitation endpoints: Early SEP parameters, combined with demographic features (age, sex), could help identify patients at risk for slow recovery or complications; (3) Optimizing treatment strategies: Providing electrophysiological rationale for adjusting or tapering medications (e.g., gabapentin, propranolol). Future research should focus on longitudinally tracking changes in SEP parameters in PSH patients and correlating them with long-term functional rehabilitation outcome measures, such as the Functional Independence Measure, to establish the specific utility of SEPs in guiding rehabilitation decisions.

This study has several limitations. First, as a retrospective single-center study, it is susceptible to selection bias. The relatively small sample size of PSH patients may affect the power of intergroup statistical comparisons, and the findings may not be fully generalizable to other clinical settings. Second, although SEP testing was performed under standardized protocols (including strict adherence to the international 10–20 system for electrode placement), measurements could still be influenced by technical factors such as individual anatomical variations and slight differences in electrode positioning. Third, while we excluded patients under the direct influence of sedatives during SEP recording, residual or prior sedative effects could potentially confound results. Future prospective studies should incorporate quantitative sedation assessment (e.g., using scales like the Richmond Agitation-Sedation Scale) as a covariate in statistical models to better control for this confounding factor. Based on the results of this study, several directions warrant future exploration. First, the predictive efficacy of SEPs for PSH needs validation in prospective, multicenter, large-sample cohorts, and their combined application value with other biomarkers (e.g., neuroimaging, inflammatory markers) should be explored. Second, longitudinal analysis linking SEP parameters to long-term functional rehabilitation outcomes (e.g., Functional Independence Measure, modified Rankin Scale) will clarify their practical clinical utility in guiding rehabilitation prognosis and personalized treatment. Furthermore, the underlying biological mechanisms for age and sex as risk factors, such as the influence of hormonal levels on the plasticity of autonomic neural circuits, require further elucidation through basic and translational research.

In summary, this study demonstrates that a reduced N20-P25 amplitude on somatosensory evoked potentials is an independent and objective electrophysiological biomarker for the development of paroxysmal sympathetic hyperactivity in patients with prolonged disorders of consciousness. Younger age and male patients are also significant risk factors for PSH. A diagnostic model combining these three predictors (N20-P25 amplitude, age, and sex) showed optimal discriminative performance. These findings provide a new basis for the early identification of PSH and risk stratification of high-risk patients, offering potential clinical value for improving management in both neurocritical care and rehabilitation settings.

## Data Availability

The original contributions presented in the study are included in the article/supplementary material, further inquiries can be directed to the corresponding author.
